# Leukocytoclastic Vasculitis: An Early Skin Biopsy Makes a Difference

**DOI:** 10.7759/cureus.7912

**Published:** 2020-05-01

**Authors:** Juan Jose Chango Azanza, Paola Michelle Calle Sarmiento, Nerea Lopetegui Lia, Swetha Ann Alexander, Viraj Modi

**Affiliations:** 1 Internal Medicine, University of Connecticut Health Center, Farmington, USA; 2 Internal Medicine, Catholic University of Cuenca, Cuenca, ECU; 3 Internal Medicine, University of Connecticut, Farmington, USA

**Keywords:** leukocytoclastic vasculitis, hypersensitivity vasculitis, small vessel vasculitis, skin biopsy, palpable purpura

## Abstract

Leukocytoclastic vasculitis (LCV) is an uncommon condition with a broad differential diagnosis. Although the clinical history, physical examination, and laboratory workup are pivotal when formulating a differential diagnosis of LCV, a skin biopsy is required in most cases to elucidate the cause. The diagnostic yield of a skin biopsy increases within the first 24 to 48 hours of the lesion onset indicating the importance of obtaining a prompt skin sample. We present the case of a 60-year-old man who presented to the emergency department with a three-day history of fevers, headaches, and a painful skin rash. He endorsed rhinorrhea and sore throat a week ago. Physical examination was notable for an erythematous papular rash with palpable violaceous purpura located mainly at the distal right leg and thigh. He also complained of painful bilateral hand edema. His complete blood count and chemistries were unremarkable. His C-reactive protein was 147 mg/L (normal value <8 mg/L), and sedimentation rate was 51 mm (normal value <15 mm). Immunoglobulin A was 509 mg/dL (normal value 82-460 mg/dL). Further workup including viral hepatitis serologies, antinuclear antibodies, complements, antineutrophil cytoplasmic antibodies, cryoglobulins, rheumatoid factor, and blood cultures yielded negative results. Therefore, it was believed that his rash was likely associated with his recent upper respiratory infection. A skin biopsy done on the first day of admission was positive for LCV without immunoglobulin A deposition. He was managed with prednisone and anti-inflammatory medications with improvement of his rash.

## Introduction

Leukocytoclastic vasculitis (LCV), also known as “hypersensitivity vasculitis,” is an uncommon condition. The incidence of cutaneous vasculitis ranges from 15.4 to 29.7 cases per million people every year. Although the clinical history, physical examination, and laboratory findings are important when formulating a differential diagnosis, a skin biopsy and dermatopathology analysis provide key information in the differentiation among the causes of cutaneous vasculitis [1]. A skin biopsy performed within the first 24 to 48 hours of lesion onset is crucial to increase the diagnostic yield when a cutaneous vasculitis is suspected [2]. We present the case of a patient who presented to the emergency room with a skin rash suspicious for a cutaneous vasculitis for whom an early punch skin biopsy performed by a dermatologist provided key information to dictate the most appropriate management. The patient was found to have an LCV and was treated with systemic steroids with remarkable improvement of his symptoms.

## Case presentation

A 60-year-old man with an unremarkable past medical history presented to the emergency department with a three-day history of fevers, headaches, and a painful skin rash. He endorsed having rhinorrhea, headaches, and sore throat a week before his presentation. He developed painful round violaceous papules at the level of his right ankle three days before coming to the hospital shortly after his fever occurred. These papules became progressively larger and coalesced into more extensive lesions that spread from his right ankle to his right thigh, abdomen, lower chest, and left lower extremity. Additionally, he developed edema located mostly on the dorsal aspect of his hands.

**Figure 1:**
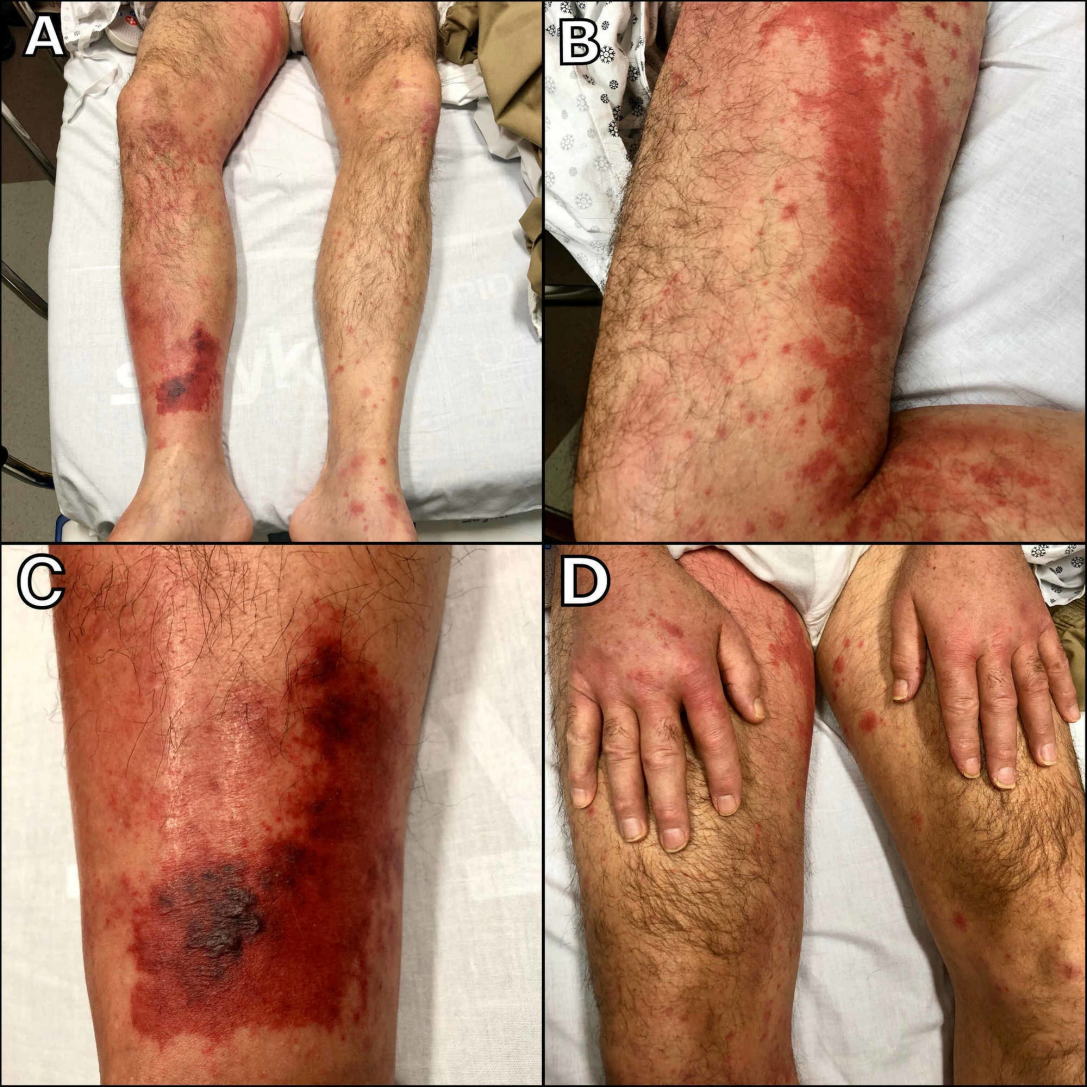
Palpable purpura located in the right leg (A) with propagation to the medial thigh (B), wine-colored vesicles (C), and bilateral hand edema (D) suggestive of cutaneous vasculitis

On physical examination, his vital signs were within normal limits except for a temperature of 38.6°C. Palpable purpura was appreciated above the medial malleolus (Figure 1A) with a chord-like purpuric lesion seen on the medial thigh that seemed to extend upwards from the malleolar lesion (Figure 1B). A closer look to the first lesion showed wine-colored vesicles with a purpuric base (Figure 1C). Bilateral dorsal hand edema was appreciated as well (Figure 1D). The rest of his examination was unremarkable.

His complete blood cell counts and chemistries were unremarkable. Inflammatory markers were elevated with a C-reactive protein of 147 mg/L (normal value <8 mg/L) and a sedimentation rate of 51 mm (normal value <15 mm). Immunoglobulin A was 509 mg/dL (normal value 82-460 mg/dL). Further workup including urine toxicology (negative for levamisole and cocaine), blood cultures, gonorrhea, chlamydia, viral hepatitis serologies, antinuclear antibodies, complements, antineutrophil cytoplasmic antibody (ANCA), cryoglobulins, and rheumatoid factor yielded negative results.

Dermatology was consulted in the emergency department and a skin biopsy was obtained in less than 24 hours from admission and less than 72 hours from the development of the rash. There was a perivascular inflammatory infiltration of neutrophils, lymphocytes, histiocytes, and eosinophils. Perivascular neutrophilic nuclear fragmentation was appreciated. Extravasated erythrocytes and nuclear dust were present in the dermis. Direct immunofluorescence revealed interstitial dermal fibrinogen deposition. IgG, IgA, IgM, and C3 were non-contributory. No pathological microorganism was found. These findings were consistent with early LCV adequately detected by this early biopsy. It was thought that the trigger for the LCV was likely a recent upper respiratory infection.

A multidisciplinary team consisting of dermatologists, rheumatologists, wound care nurses, the primary medicine team, and others was involved in this patient's care. Given the systemic symptoms including fevers, headaches, and a diffuse rash, the patient was administered systemic corticosteroids with excellent response in his rash and hand edema. Pain control was achieved with acetaminophen and ibuprofen. His rash progressively improved and his fevers subsided within the first five days of treatment. After a multidisciplinary discussion with the patient, it was decided that immunosuppressive therapy was not necessary at the time of discharge. The patient’s rash resolved completely after discharge.

## Discussion

Vasculitis is defined as the inflammation of the blood vessels casing wall compromise with subsequent hemorrhage and/or ischemia [3]. LCV or “hypersensitivity vasculitis” is an uncommon condition. The incidence of cutaneous vasculitis ranges from 15.4 to 29.7 cases per million people every year [1]. LCV affects more females than males and is more common in adults than in children. Children are more likely to develop Henoch-Schönlein purpura. Up to half of the cases are idiopathic and potential causes include infection, medications, lymphoproliferative disorders, inflammatory diseases, connective tissue diseases, and malignancy [4].

The pathogenesis of LCV is related to immune complex formation generally of immunoglobulins M and G with activation of the complement cascade. The liberation of chemotactic substances with increased expression of leukocyte adhesion molecules causes a migration of neutrophils which produces an inflammatory response with damage to the blood vessels (vasculitis) with degranulation and death of neutrophils (leukocytoclasia) [4]. The degradation of the polymorphonuclear cells causes a release of nuclear debris that is described histologically as “nuclear dust.” The blood vessels experience increased permeability with leakage of fluids and cells into the extravascular space. Fibrinoid necrosis with an accumulation of plasma proteins that are converted into fibrin products is a typical finding of vasculitis and is seen in most early vasculitic lesions [3].

The clinical manifestations include palpable purpura, petechiae, and other skin lesions. Palpable purpura can be seen in up to 64% of patients, petechiae and purpuric macules in 36%, vesicles and bullae in 16%, ulcers in 9.3%, subcutaneous nodules in 2.7%, and edema in hands and feet in 1.3% of patients [5]. The most common site is the lower extremities particularly above the ankles [1,3]. Systemic involvement can be seen in up to 50% of patients with alteration of the kidneys, muscles, joints, lungs, gastrointestinal organs, central nervous system, and other systems [4]. Most cases are idiopathic. However, important triggers include infection and medications (hypersensitivity vasculitis). Other important causes include lymphoproliferative disorders, inflammatory diseases, connective tissue diseases, and malignancy [4].

The differential diagnosis for LCV is extensive. The main differentials considered in this case were small-vessel vasculitides and infection. Immunoglobulin A vasculitis can have a similar presentation but occurs more frequently in children aged less than 10 years, affects the kidneys and gastrointestinal tract, and would show a prominent immunoglobulin A deposition appreciated on skin biopsy. Urticarial vasculitis would be recurrent and present with low complement levels when there is systemic involvement. Microscopic polyangiitis typically would have ANCA-positive antibodies (about 10% of cases may be ANCA negative) and renal involvement in most cases. Granulomatosis with polyangiitis presents with sinusitis, nasal and oral ulcerations, or any other type of upper airway involvement, ANCA-positive antibodies in most cases and renal involvement. Eosinophilic granulomatosis with polyangiitis shows the classic triad of asthma, rhinitis, and peripheral blood eosinophilia with ANCA-positive antibodies in 40%-60% of cases. Other causes including arthropod bites, cocaine levamisole toxicity, thromboangiitis obliterans, among others. Infectious causes such as bacterial (cellulitis, impetigo), viral (herpes), and fungal were considered but palpable purpura with an extensive distribution of the lesions would not be seen with these types of infections. The differential diagnosis can be challenging, especially between small-vessel vasculitides. Therefore, a biopsy is crucial to reach a definitive diagnosis. It was thought that the trigger for the LCV was likely a recent upper respiratory infection.

A skin biopsy with direct immunofluorescence is crucial to confirm the diagnosis and determining if patients are at high risk of systemic complications [6]. For the diagnosis of any form of cutaneous vasculitis, a punch biopsy is adequate as it permits the analysis of the entirety of the dermis. Multiple authors agree that a biopsy should be performed early to increase the diagnostic yield which is usually between 24 and 48 hours after the lesion onset [1,2,4,6]. A second biopsy with direct immunofluorescence may be helpful if a first biopsy was inconclusive [7]. In our case, the biopsy was done soon after the first contact with the patient which was before 72 hours of symptom onset. Although the ideal time for a biopsy of the skin would have been less than 48 hours, his biopsy was done early enough to detect the characteristic findings of LCV. Therefore, it is recommended to contact dermatology or any provider with expertise in obtaining skin biopsies promptly. The selection of the site is important and having an expert opinion with an interpretation of which lesions seem “fresh” can help to increase the chances to find immune complex deposits and other findings suggestive of vasculitis [6].

The treatment depends on multiple factors including the cause, systemic involvement, and complications. If the cause is a modifiable factor, the focus should be addressing the issue such as stopping a culprit medication or treatment of infection if feasible. General measures should be recommended such as avoiding prolonged standing, avoidance of cold exposure, wearing loose clothing, and leg elevation, keeping the extremities warm. Symptomatic management of pain and pruritus can be achieved with antihistamines and non-steroidal anti-inflammatory drugs (NSAIDs). It is important to understand that these recommendations do not alter the course of the disease or prevent recurrence [8]. More than half of the patients with cutaneous vasculitis only do not require systemic therapy other than symptomatic management with NSAIDs [9]. Systemic involvement warrants treatment with systemic corticosteroids, colchicine, dapsone, or immunosuppressant agents when refractory or recurrent cases occur [10-13].

## Conclusions

LCV is a rare condition that classically presents with a rash located in the lower extremities with palpable purpura. When LCV is suspected, it is crucial to obtain a skin biopsy within 24 to 48 hours of symptom onset to increase the diagnostic yield. Involving a team with expertise in performing skin biopsies such as dermatology is pivotal to aid in the diagnosis and management of this condition.
